# Metabolomic profiling identifies metabolites in the pheromone glands of *Agriophara rhombata* associated with the synthesis and release of female pheromone signals

**DOI:** 10.1016/j.heliyon.2024.e40768

**Published:** 2024-11-28

**Authors:** Hao Qu, Yaqin Long, Long Chen, Ziwen Luo, Hongyun Chen, Xuesong Wang, Lixue Long, Jun Tian, Tingting Jing, Linbo Chen

**Affiliations:** a Tea Research Institute, Yunnan Academy of Agricultural Sciences, Kunming, 650000, China; b State Key Laboratory of Tea Plant Biology and Utilization, International Joint Laboratory on Tea Chemistry and Health Effects, Anhui Agricultural University, 230036, Hefei, Anhui, China; c Yunnan Provincial Key Laboratory of Tea Science, Menghai, 666201, China; d Kunming Colourful Yunnan King-shine Tea Industry Co., Ltd, Kunming, 650500, China

**Keywords:** Tea moth, Pheromone glands, Metabolomic profiling, Sex pheromones

## Abstract

The tea moth pest, *Agriophara rhombata* is an economically important and highly damaging pest that drastically affects tea plant leaves. The chemical composition of its pheromone glands metabolites before and during calling period has not been reported yet. Therefore, the present study aimed at the metabolomic profiling of female moths’ sex pheromones glands before and during calling period using gas chromatography time-of-flight mass spectrometry. A total of 114 significant differentially expressed metabolites were identified including 54 up- and 70 down-regulated metabolites in pheromone glands of the female moth. Two of the important previously recognized moth pheromones were identified including E,Z-5,7-dodecadien-1-ol acetate and Z-12-Octadecen-1-ol acetate, which were downregulated. The top ten up-regulated metabolites were “dodecanamide”, “tetradecanamide”, “2-propyn-1-amine, N,N-dimethyl”, “cyclohexane, (1-methylethyl)”, “tetradecane, 2-methyl”, “1-cyclopentyleicosane”, “cyclohexane, octyl”, “1-decan-3-one”, “cyclopentane, decyl” and “cyclopentadecane”. In conclusion, while most of the identified compounds have not previously been identified as primary pheromones in moths, their differential expression in *A. rhombata's* pheromone glands during the calling period strongly suggests their supporting roles in the synthesis, stabilization, or release of the active pheromone components.

## 1 Introduction

Insects having chemoreceptors which are frequently found in antennae, are used to perceive a variety of ambient compounds, including volatile and non-volatile odorants [1]. This sensory system helps to obtain information about possible oviposition sites, food supplies, the social milieu, and ideal partners for reproduction [2]. The olfactory function of antennae is essential to the courting behavior of insects, in which an adult male uses species-specific sex pheromones emitted by a conspecific female to discover and hunt for a mate [3]. The antennae of male moths are delicately designed to recognize and differentiate even the smallest of these sex pheromones among mixture of other scents. Its olfactory receptor neurons can detect individual pheromone molecules as well as intermittent pheromone filaments of highly fluctuating frequencies up to around 30 Hz over a broad range of concentrations [4]. Lepidoptera insects are classified into around 160,000 species [5], along with approximately 700 different Lepidoptera pheromones that have been found [6] since the identification and structural characterization of the first sex-related pheromone *Bombyx mori*, a silkworm moth [7]. Their primary characteristics, such as species-specificity, non-toxicity to mammals and other beneficial organisms, effective at very low concentration, and quick environmental degradation [8].

Agriophara *rhombata* Meyr, commonly named as tea grain moth or tea ash wood moth belongs to Lepidoptera order and family Depressariidae. *Agriophara rhombata* is an invasive pest that damages tea plant leaves [9]. The tea moth has been a major pest in India, now extended its geographical range, and is documented in various provinces of China including Taiwan, Fujian, Yunnan, Hainan, and Guangdong [9]. Many of the moth species are important in agriculture and they extensively rely on pheromones to facilitate mating behaviors. Moth pheromones are categorized into three primary groups including Type I, Type II, and Miscellaneous Pheromones. These groups are distinguished by their distinct chemical compositions and biological roles. The most prevalent class of sex pheromones in moths are type I pheromones. Usually, they are long-chain unsaturated aliphatic molecules such as acetates, alcohols, and aldehydes. These pheromones are widely distributed in many moth species and are utilized by females to attract males for mating [10].

Sex pheromones are produced in specialized pheromone glands located on the eighth and ninth abdominal segments of female moths, and are made up of specific combinations of volatile substances, mostly straight-chain primary alcohols (C_10_–C_18_), aldehydes, ketones, isoprenoids, lactones, esters, and triacylglycerides [11]. Even though many species share identical pheromone molecules, the precise components and their ratios in the pheromone mixture serve as surprisingly accurate, species-specific signals for mating [12]. Sex pheromones include courtship pheromones, which cause a range of close-range reactions in the insect partner, and sex attractant pheromones, which cause upwind oriented movements to the conspecific individual [13].

Metabolomics entails the systematic detection and quantification of the metabolites and their variations respective to time in biological samples. Liquid chromatography mass spectrometry (LC-MS) and gas chromatography mass spectrometry (GC-MS) are effective analytic techniques for unveiling thousands of metabolites as insect sex pheromones. The chemical attributes of the targeted compounds and their concentrations in the samples play a role in the choice of analytical technique [6]. This study aimed to identify the essential components of the sex pheromones glands metabolites before and during calling period in female moths. To investigate the metabolites in pheromone glands samples, metabolomics analysis employing gas chromatography combined with time-of-flight mass spectrometry (GC-TOFMS) was adopted to compare the metabolic changes from the female moth pheromone glands before and during the calling period.

## 2 Materials and methods

The larvae of moths (*Agriophara rhombata*) were collected from tea trees (*Camellia sinensis*) in Pu'er City, Yunnan Province, China (longitude: 100.9 East, latitude: 22.7 North) and were cultured in the laboratory on wheatgerm-casein diet at 25 ± 1 °C, 16:8 light: dark photoperiod and 65 ± 5 % relative humidity until they pupated. Then pupae were sexed and kept separately. For sexing pupae in moths, the first step was to look at the external genital papillae (small protrusions) on the ventral side of the pupae. When sexing female pupae, the genital papillae are located on the 8th abdominal segment and typically appear as a single slit, while for male pupae, they are located on the 9th segment and typically appear as two small slits or pores.

The pupae were checked daily for emergence and supplied with 10 % honey solution as food for the emerging adults. The sexual maturity of *A. rhombata* females typically occurs within 1–2 days post-emergence. The pheromone release rhythm in these moths is tightly regulated and generally coincides with the calling period, which occurs at the end of the scotophase (the dark period of the light-dark cycle). During this time, the females exhibit calling behavior, characterized by the extrusion of pheromone glands, which facilitates the release of sex pheromones to attract males. After adult emergence, female moths were placed in individual containers, each provided with a small tea tree leaf as an oviposition substrate. The containers were maintained at the same environmental conditions (25 ± 1 °C, 16:8 light photoperiod, and 65 ± 5 % relative humidity). Females were checked every 12 h for oviposition activity. Oviposition data, including the number of eggs laid per female and the timing of oviposition relative to scotophase, were recorded over three days post-emergence. Proportions of oviposition across time periods were calculated for analysis. We divided the experiment in two groups, Control group (control): pheromone glands samples from moths before calling period; and treatment group (Gd): pheromone glands samples from moths during calling (at the end of the scotophase). The pheromone glands in female moth were squeezed out from the abdomen using forceps from both groups during the specified periods for each group. Females moth were cooled to −20 °C for at least 5 min prior to gland excision. Pheromone glands were extracted in hexane for 2 h (ca. 10 μL of hexane per abdominal tip) [14]. Extracts were stored at −20 °C until analysis. A total of 20 pheromone glands were pooled to form one sample. Each group (control and Gd) had six samples (replicates). Later the GC-TOFMS analysis of extracted samples was performed.

### 2.1 Gas chromatography time-of-flight mass spectrometry (GC-TOF-MS) analysis for metabolites

SHIMADZU GC2030-QP2020 NX gas chromatography-mass spectrometer was used for analysis. The system utilized a DB-5MS capillary column. 1 μL aliquot of sample was injected in splitless mode. Helium was used as the carrier gas, the front inlet purge flow was 3 mL min^−1^, and the gas flow rate through the column was 1 mL min^−1^. The initial temperature was kept at 50 °C for 1 min, then raised to 310 °C at a rate of 10 °C min^−1^, then kept for 11.5 min at 310 °C. The injection, transfer line, and ion source temperatures were 280, 280 and 200 °C, respectively. The energy was −70 eV in electron impact mode. The mass spectrometry data were acquired in full-scan mode with the *m*/*z* range of 50–500 at a rate of 12.5 spectra per second after a solvent delay of 7.2 min.

### 2.2 Data processing and multivariate statistical analysis

The compounds were identified using the National Institute of Standards and Technology (NIST) Mass Spectral Library (Version 2.3) for spectral matching. Compound identification was based on a match factor greater than 85 % with the library spectra. No co-injection was performed for compound confirmation. The LECO ChromaTOF software was used for peak deconvolution and identification of metabolites from the total ion chromatogram. Using LECO ChromaTOF software (version 4.44), the raw data obtained from GC-TOF-MS were first converted into NetCDF (∗.cdf) format. Following this conversion, the metAlign software program was used to process the NetCDF files. Later on, peak detection, alignment, and retention time correction were performed. The data were then processed and used to perform principal component analysis (PCA) using SIMCA-P+12.0, a software program (Umetrics in Umea, Sweden). Volcano plot analysis was performed based on the fold changes and p-values to screen the metabolites in MetaboAnalyst (Version 5.0). Hierarchical cluster heat map was also generated in MetaboAnalyst (Version 5.0) to identify the enriched metabolites.

Statistical analysis of activities of moth emergence was conducted through one way analysis of variance (ANOVA).

## 3 Results

### 3.1 Behavioral sequences after emergence

**Fig. 1:**
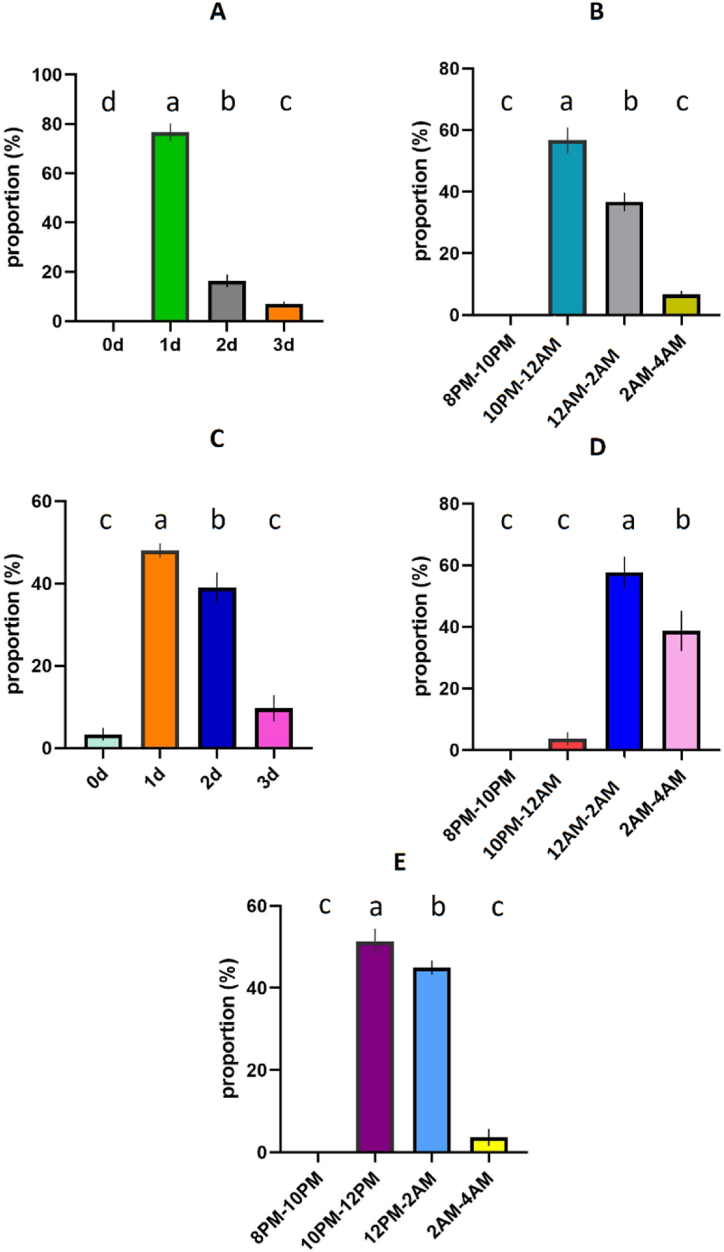
Tea moth activities after emergence; **(A)** courtship activity (days) **(B)** courtship activity (day hours), **(C)** the oviposition activity (days), **(D)** oviposition activity (day hours), and **(E)** the mating activity during the day hours observed throughout the experiment. Different letters above the bars show significant difference at *p* < 0.05.

*Agriophara rhombata* tea moth behavioral sequences after emergence were categorized into following phases: courtship activity after emergence (days), courtship activity after emergence (day hours), oviposition activity after emergence (days), oviposition activity after emergence (day hours), and mating time during day hours (Fig. 1A–E; Supplementary Table S1). Results showed that the courtship activity was higher during day 1 after emergence (about 75 %) (Fig. 1A) during 10:00PM to 12:00AM (about 55 %) (Fig. 1B). Similarly, the oviposition activity was also higher during day 1 (about 50 %) (Fig. 1C) during 12:00AM to 2:00AM (about 55 %) (Fig. 1D). Further, the higher mating time was observed during10:00PM to 2:00AM (about 95 %) (Fig. 1E).

### 3.2 The principal component analysis

**Fig. 2:**
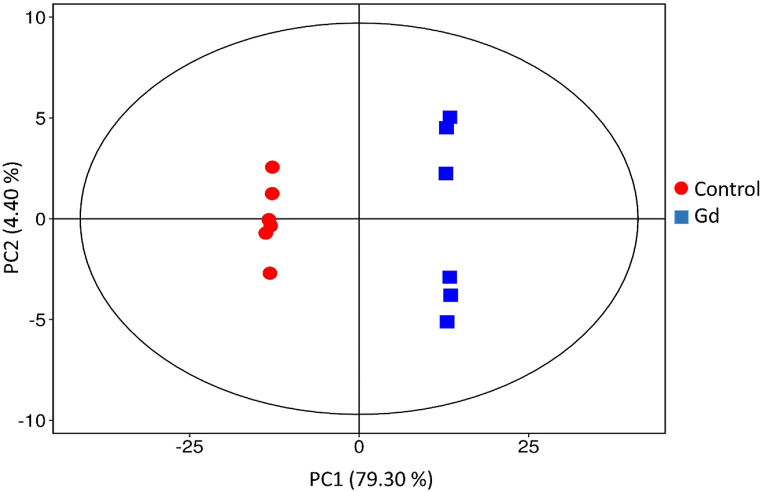
Principal component analysis (PCA) shows the differences and similarities among groups and the distribution of samples in each group (Control and Gd). In PCA plot, each point represents different samples and the distance among them shows the extent of differences. Both group samples are differently colored.

The principal component analysis (PCA) was performed to unveil the differences and similarities between groups and group samples (Control & Gd) based on their volatile profiles (Fig. 2). A PCA plot was constructed to reduce the dimensionality of a large dataset, thereby elucidating the variations within the data as two components (PC1 & PC2). PC1 explained 79.3 % of total variance, whereas PC2 explained 4.4 % of the total variance. Results showed both groups (Control & Gd) clearly separated apart respective to their components. Further, the control group samples exhibited more similar metabolites profiles as they were closely clustered compared to Gd group samples that were distantly clustered demonstrating that these samples had diverse volatile profiles.

### 3.3 Differential metabolite analysis

**Fig. 3:**
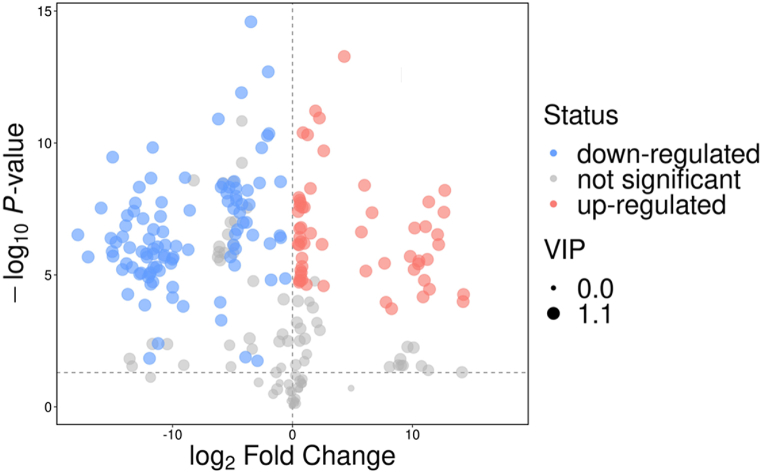
Volcano plot showing the differentially expressed metabolites between two groups (Control vs Gd). Each point in volcano plot represents a metabolite (volatile compound) with red points displaying the significantly up-regulated metabolites, while blue points displaying the significantly down-regulated metabolites.

Differential metabolite analysis was performed to identify the significantly differentially expressed metabolites in both groups (Fig. 3). The annotated metabolites were screened based on the VIP score and fold change to identify the substantially important metabolites in both groups. A total of 114 significant metabolites (p ≤ 0.05) were identified including fifty-four up-regulated metabolites while seventy down-regulated metabolites (Supplementary Table S2). Two of the identified compounds including E,Z-5,7-dodecadien-1-ol acetate and Z-12-Octadecen-1-ol acetate, has been previously recognized as moth pheromones were downregulated.

**Fig. 4:**
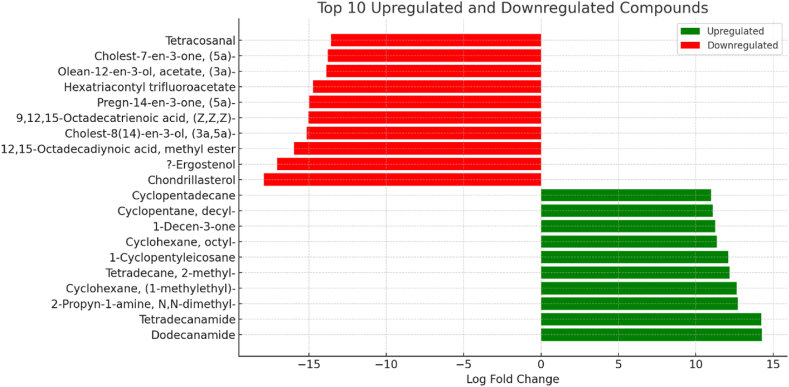
Top ten up-regulated and down-regulated volatiles in two groups (Control vs Gd).

The top ten significantly up-regulated metabolites were related to classes amides (“dodecanamide” and “tetradecanamide”), amines (“2-propyn-1-amine, N,N-dimethyl”), hydrocarbons (“cyclohexane, (1-methylethyl)”, “tetradecane, 2-methyl”, “1-cyclopentyleicosane”, “cyclohexane, octyl”,“cyclopentane, decyl”, and “cyclopentadecane”), and ketone (“1-decan-3-one”), while sterols and steroids (“chondrillasterol”, “ergostenol”, “cholest-8(14)-en-3-ol, (3a,5a)”, “pergn-14-en-3-one, (5a)”, “cholest-7-en-3-one, (5a)”, and “olean-12-en-3-ol, acetate, (3a)”), fatty Acids and derivatives (“12, 15-octadecadiynoic acid, methyl ester”, and “9,12,15-octadecatrienoic acid, (Z,Z,Z)”), ester compounds (“hexatriacontyl trifluoroacetate” and “tetracosanal”) were top ten significantly down-regulated metabolites in both groups (Control & Gd) (Fig. 4).

## 4 Discussion

The oviposition behavior in *A. rhombata* aligns with nocturnal moth patterns, with peak egg-laying occurring shortly after emergence, particularly between 12 a.m. and 2 a.m., coinciding with pheromone release during the scotophase [15]. This early reproductive investment is typical in moths to maximize fertilization chances. The decline in oviposition after the first day likely reflects energy trade-offs, as seen in other species. Environmental factors like temperature, light cycles, and food availability also play crucial roles in oviposition behavior [16]. Similar patterns have been observed in moths like *Helicoverpa armigera* and *Spodoptera frugiperda* [17,18].

The Lepidoptera order has offered important insights into the intricate systems that regulate insect communication and reproduction. The GC-TOF-MS analysis of pheromone glands before and during the calling period of this species has revealed two important compounds E,Z-5,7-dodecadien-1-ol acetate and Z-12-Octadecen-1-ol acetate, which have previously been recognized as moth pheromones [19,20]. The E,Z-5,7-dodecadien-1-ol acetate was detected using the electroantennogram technique in pine moth (*Dendrolimus spectabilis* Butler), that is harmful defoliator of pine trees [21]. Both of these pheromones were downregulated in the present study that could be due to the sampling time as samples were taken before calling and during calling (at end of scotophase) [22].

In this study, GC-TOF-MS analysis enabled the identification of several key compounds that may potentially involved in pheromone signaling in female moths. Compounds such as “dodecanamide”, “tetradecanamide”, “2-propyn-1-amine, N,N-dimethyl”, “cyclohexane, (1-methylethyl)”, “tetradecane, 2-methyl”, “1-cyclopentyleicosane”, “cyclohexane, octyl”, “1-decan-3-one”, “cyclopentane, decyl”, and “cyclopentadecane” were identified based on their mass spectra and retention times, providing a foundation for understanding the chemical ecology of this species. While GC-TOF-MS is a powerful tool for identifying chemical compounds, it does not confirm the biological activity of these compounds in insect communication. Therefore, although the chemical profiles are suggestive of pheromone activity, further studies are required to confirm whether these compounds elicit a physiological response in the moth antennae. However, their differential expression in pheromone glands shows that they might be involved in the synthesis or release of moth pheromones.

Amides such as dodecanamide and tetradecanamide, which have not traditionally been classified as pheromones, have been found in the cuticles and pheromone glands of several insects. Amides can help pheromone compounds maintain their structural integrity and allow for more regulated release [23]. In the case of *A. rhombata*, the increased abundance of these amides throughout the calling phase might imply their role in stabilizing pheromone blends or as intermediates in the biosynthetic process that leads to the synthesis of more volatile, active pheromone components. 2-Propyn-1-amine, N,N-dimethyl, is an amine and propargylamine derivative. Amines have been demonstrated in moths to aid in the production of pheromone chemicals, frequently by aiding the alkylation and acylation steps required to produce the final pheromone structure [24]. Hydrocarbons such as tetradecane, 2-methyl, cyclohexane, (1-methylethyl), 1-cyclopentyleicosane, and cyclohexane, octyl are well-known components of insect cuticles, where they function as desiccation barriers and contact pheromones [25]. Hydrocarbons are frequently found in pheromone mixtures used by moths to communicate species-specific signals. The increased expression of these hydrocarbons in *A. rhombata* during the calling phase might indicate their function in modifying the volatilization or dispersion of pheromone components, hence increasing the efficacy of the pheromone signal. The identification of ketones, such as 1-decan-3-one, have been found in the pheromone glands of many insects, where they frequently act as intermediates or byproducts in pheromone production [26]. The higher levels of 1-decan-3-one in *A. rhombata* during the calling phase may indicate its participation in the last stages of pheromone production or in the activation of enzymes that allow pheromone release from the glands [27]. The discovery of hexadecane, 2-methyl and undecane, 2,8-dimethyl as differentially expressed molecules throughout the calling phase is very intriguing. These branched hydrocarbons, while not pheromones, might impart synergistic effects in the pheromone blend, increasing the overall attractiveness of the signal to potential mates [27]. Furthermore, the structural similarities between these molecules and known pheromone hydrocarbons indicate that they might be involved in the pathways involved in the synthesis of active pheromone components [23].

One limitation of this study is the absence of GC-EAD (Gas Chromatography-Electroantennographic Detection) analysis, which is commonly used to confirm that the identified compounds are biologically active pheromones by testing the electrophysiological response of the moth antennae. This technique is critical in establishing the biological relevance of the compounds detected by GC-MS. Future studies could be aimed to address this limitation by conducting GC-EAD experiments to verify the pheromonal activity of the identified compounds. Additionally, behavioral assays could be performed to observe the attraction or behavioral responses of moths to synthetic versions of these compounds.

## 5 Conclusions

In conclusion, while most of the identified compounds have not previously been identified (except for E,Z-5,7-dodecadien-1-ol acetate and Z-12-Octadecen-1-ol acetate) as primary pheromones in moths, their differential expression in *A. rhombata's* pheromone glands during the calling period strongly suggests their supporting roles in the synthesis, stabilization, or release of the active pheromone components. However, further studies including functional assays and behavioral investigations, are essentially required to determine the specific roles of these chemicals in the pheromone communication system of *A. rhombata.*

## CRediT authorship contribution statement

**Hao Qu:** Writing – original draft, Visualization, Methodology, Formal analysis, Data curation, Conceptualization. **Yaqin Long:** Validation, Formal analysis, Data curation. **Long Chen:** Methodology, Formal analysis, Data curation. **Ziwen Luo:** Software, Formal analysis, Data curation. **Hongyun Chen:** Methodology, Investigation, Formal analysis. **Xuesong Wang:** Validation, Software, Investigation. **Lixue Long:** Validation, Resources, Data curation. **Jun Tian:** Resources, Methodology, Formal analysis. **Tingting Jing:** Writing – review & editing, Visualization, Supervision, Resources, Project administration, Data curation, Conceptualization. **Linbo Chen:** Writing – review & editing, Visualization, Project administration, Methodology, Investigation, Funding acquisition, Formal analysis, Data curation, Conceptualization.

## Ethics and consent declarations

This study was reviewed and approved by [full name of approving ethics committee] with the approval number: [approval number], dated [date of ethics approval].

## Data availability statement

Data has been uploaded to “Metabolights” with accession number MTBLS10611.

## Funding

This research was supported by the Agricultural Joint Special Project of Yunnan (202101BD07001-029), the 10.13039/501100001809National Natural Science Foundation of China (32160724) and The Open Fund of 10.13039/501100015016State Key Laboratory of Tea Plant Biology and Utilization (SKLTOF20200117).

## Declaration of competing interest

The authors declare that they have no known competing financial interests or personal relationships that could have appeared to influence the work reported in this paper.
