# Supplementary material for: Metabolomic profiling identifies metabolites in the pheromone glands of Agriophara rhombata associated with the synthesis and release of female pheromone signals
Source: Heliyon. 2024 Nov 28;10(23):e40768. doi: 10.1016/j.heliyon.2024.e40768 (PMC11648114; doi:10.1016/j.heliyon.2024.e40768)
Supplement: Multimedia component 1 [file mmc1.docx]

**Supplementary Table 1. Tea moth (*A. rhombata*) activities after emergence**

| **(A) Proportion of courtship activity (days) (%)** | | | |
| --- | --- | --- | --- |
| Day 0 | Day 1 | Day 2 | Day 3 |
| 0±0.00^d^ | 76.67±351^a^ | 16.33±2.51^b^ | 7±1.00^c^ |
| **(B) Proportion of courtship activity (day hours) (%)** | | | |
| 8PM-10PM | 10PM-12PM | 12PM-2AM | 2AM-4AM |
| 0±0.00^c^ | 56.67±4.16^a^ | 36.67±3.05^b^ | 6.67±1.16^c^ |
| **(C) Proportion of the oviposition activity (days) (%)** | | | |
| Day 0 | Day 1 | Day 2 | Day 3 |
| 3.33±1.53^c^ | 48±1.73^a^ | 39±3.61^b^ | 9.66±3.21^c^ |
| **(D) Proportion of oviposition activity (day hours) (%)** | | | |
| 8PM-10PM | 10PM-12PM | 12PM-2AM | 2AM-4AM |
| 0±0.00^c^ | 3.67±2.08^c^ | 57.67±5.13^a^ | 38.67±6.51^b^ |
| **(E) Proportion of the mating activity (day hours) (%)** | | | |
| 8PM-10PM | 10PM-12PM | 12PM-2AM | 2AM-4AM |
| 0±0.00^c^ | 51.33±3.06^a^ | 45±1.73^b^ | 3.67±2.08^c^ |

Letters (a, b, c, and d) describes the significance levels at p-value 0.05. Each Treatment group was repeated three times (three replicates)
